# Gene number determination and genetic polymorphism of the gamma delta T cell co-receptor WC1 genes

**DOI:** 10.1186/1471-2156-13-86

**Published:** 2012-10-16

**Authors:** Chuang Chen, Carolyn TA Herzig, Leeson J Alexander, John W Keele, Tara G McDaneld, Janice C Telfer, Cynthia L Baldwin

**Affiliations:** 1 Department of Veterinary and Animal Sciences, University of Massachusetts, Amherst, MA, 01003, USA; 2 Department of Agriculture, Fort Keogh Livestock & Range Research Laboratory, USDA-ARS Fort Keogh LARRL, Miles City, MT, 59301, USA; 3 USDA-ARS, U. S. Meat Animal Research Center, Clay Center, NE, 68933, USA; 4 Current Address: Department of Epidemiology, Mailman School of Public Health, Columbia University, New York, NY, 10032, USA

**Keywords:** Bovine, WC1, γδ T cells

## Abstract

**Background:**

WC1 co-receptors belong to the scavenger receptor cysteine-rich (SRCR) superfamily and are encoded by a multi-gene family. Expression of particular WC1 genes defines functional subpopulations of WC1^+^ γδ T cells. We have previously identified partial or complete genomic sequences for thirteen different WC1 genes through annotation of the bovine genome Btau_3.1 build. We also identified two WC1 cDNA sequences from other cattle that did not correspond to sequences in the Btau_3.1 build. Their absence in the Btau_3.1 build may have reflected gaps in the genome assembly or polymorphisms among animals. Since the response of γδ T cells to bacterial challenge is determined by WC1 gene expression, it was critical to understand whether individual cattle or breeds differ in the number of WC1 genes or display polymorphisms.

**Results:**

Real-time quantitative PCR using DNA from the animal whose genome was sequenced (“Dominette”) and sixteen other animals representing ten breeds of cattle, showed that the number of genes coding for WC1 co-receptors is thirteen. The complete coding sequences of those thirteen WC1 genes is presented, including the correction of an error in the *WC1-2* gene due to mis-assembly in the Btau_3.1 build. All other cDNA sequences were found to agree with the previous annotation of complete or partial WC1 genes. PCR amplification and sequencing of the most variable N-terminal SRCR domain (domain 1 which has the SRCR “a” pattern) of each of the thirteen WC1 genes showed that the sequences are highly conserved among individuals and breeds. Of 160 sequences of domain 1 from three breeds of cattle, no additional sequences beyond the thirteen described WC1 genes were found. Analysis of the complete WC1 cDNA sequences indicated that the thirteen WC1 genes code for three distinct WC1 molecular forms.

**Conclusion:**

The bovine WC1 multi-gene family is composed of thirteen genes coding for three structural forms whose sequences are highly conserved among individual cattle and breeds. The sequence diversity necessary for WC1 genes to function as a multi-genic pattern recognition receptor array is encoded in the genome, rather than generated by recombinatorial diversity or hypermutation.

## Background

Workshop cluster 1 (WC1) co-receptors belong to group B of the scavenger receptor cysteine-rich (SRCR) superfamily, as do CD163, CD5, CD6, and Spα, all of which are expressed in immune system cells [1]. We have shown that WC1 is a member of the CD163 multigene family whose other members are CD163A, CD163b and CD163c-α [2]. WC1 co-receptors are composed of up to eleven extracellular SRCR domains with interdomain homology, organized in the domain pattern of a-[b-c-d-e-d]-[b-c-d-e-d’] according to the nomenclature of Sarrias et al. [1]. The greatest difference among WC1 genes occurs in the most distal SRCR domain (“a” pattern) with identities as low as 50%, contrasting with other SRCR domains which have identities of approximately 90% with like domains [3]. WC1 and CD163c-α have the most similar extracellular SRCR domain organization [3,4] and it has been proposed that the human and murine homologs of ruminant WC1 are CD163c-α (known as SCART1 and SCART2 and also expressed on γδ T cells in mice) [2,5,6]. We have shown that a multigenic array of WC1/CD163c-α homologues is conserved over evolutionary time including in the prototherian mammal duck-billed platypus and in the sauropsid chicken [2].

Based on reactivity with specific monoclonal antibodies (mAbs) using WC1-transfected cells, WC1 bearing γδ T cells (WC1^+^ γδ T cell) were defined as WC1.1^+^, WC1.2^+^, and WC1.3^+^ wherein the WC1.3^+^ population is a subpopulation of WC1.1^+^ cells [7]. The WC1.1^+^ and WC1.2^+^ mAb-defined subpopulations are largely nonoverlapping and may be functionally distinct subsets of WC1^+^ γδ T cells since they have different cytokine production and cellular proliferation in response to stimulation [8,9]. For example, *ex vivo* WC1.1^+^ γδ T cells, but not WC1.2^+^ γδ T cells, proliferate well to the γδ T cell antigens of *Leptospira*, and produce IFN-γ in response to either antigen or IL-12 [8,9]. However, WC1.2^+^ γδ T cells respond to the rickettsiales bacteria *Anaplasma*[10]. It was also notable that WC1.1^+^ cells decreased steadily with aging, while the WC1.2^+^ cells did not, suggesting their different functional roles [9]. Since γδ TCR gene usage is not different between WC1.1^+^ and WC1.2^+^ γδ T cells [11], this may suggest that expression of particular WC1 family members directs the antigen-specific activation of γδ T cells.

Based on Southern blot analysis, it was predicted that there were over fifty WC1 (also known as T19) ovine genes [12,13], and nineteen WC1 bovine genes [14]. To better characterize the WC1 co-receptor family, we annotated the WC1 regions in the bovine genome Btau_3.1 assembly, identifying partial or complete sequences of thirteen WC1 genes distributed between two regions on chromosome 5 [3]. The annotated number of WC1 genes is consistent with our previous study that identified thirteen different WC1 intracytoplasmic tail transcripts [15] but was fewer than the nineteen genes predicted by Southern blot analysis. In addition, we had also identified two additional Domain A transcript sequences, *WC1-nd1* and *WC1-nd2*, derived from a different breed of cattle than that used for the genome sequencing [3]. The missing genomic evidence for *WC1-nd1* and *WC1-nd2* in the genome of the animal “Dominette” could be due to gene number variation, polymorphisms among individual cattle or alternatively gaps in the assembled genome. Thus, the complexity of the WC1 multi-gene family remained unresolved including gene number and potential sequence polymorphisms; more recent assemblies have not ameliorated these problems.

Real-time quantitative PCR (Q-PCR) is highly sensitive and allows quantification of very small changes in sequence and rare transcripts [16,17]. Real-time Q-PCR has evolved to increase the accuracy and efficiency of the nucleic acid quantification process, making Q-PCR a reliable and powerful tool [18]. For example, Q-PCR has successfully quantified viral copy number and gene number in transgenic animals and measured oncogene amplification in tumor cells [19-23]. In relative quantification methods, the amount of target gene in a sample is presented relative to a calibrator which contains both target and reference genes at a constant ratio [24]. In this study, we adapted it to determine the gene number of WC1 genes in bovine genomes.

## Methods

### PBMC

Cattle of the Belted Galloway and Holstein breeds were 12–24 months of age. Blood was collected into heparin by venipuncture of the jugular vein. Peripheral blood mononuclear cells (PBMC) were isolated from blood via density gradient centrifugation over ficoll-hypaque (Ficoll-Paque, LKB-Pharmacia Biotechnology, Piscataway, NJ) using standard techniques and viable cell concentrations determined by trypan blue exclusion. PBMC were cultured at 2.5 × 10^6^ cells/ml with Concanavalin A (ConA; 1.0 μg/ml; Sigma-Aldrich, St. Louis, MO) or leptospira antigen ( [9], 0.5 μg/ml; sonicated whole cells of *L. borgpetersenii* serovar hardjo clone RZ33) in RPMI 1640 medium containing 10% heat-inactivated fetal bovine serum (HyClone, Logan, UT), 2 mM L-glutamine, 50 μM 2-mercaptoethanol and 50 μg/ml gentamicin at 37°C with 5% CO_2_ in air for six days. All animal use complied with federal guidelines and had Institutional Animal Care and Use Committee (IACUC) approvals.

### Genomic DNA extraction and RNA isolation

Genomic DNA of seven cattle from two different breeds (5 Belted Galloway and 2 Holstein) was extracted from whole blood using FlexiGene DNA Kit (50) (Qiagen, Valencia, CA) according to the manufacturer’s protocol at the University of Massachusetts. To isolate RNA, pelleted ex vivo, ConA-activated, and *Leptospira*-activated PBMC, as well as sorted WC1.1^+^ γδ T cells, were resuspended in TRIzol (Invitrogen, Carlsbad, CA) and RNA was isolated according to the manufacturer’s protocol. Reverse transcription (RT) was performed using 1 μg of total RNA, oligo dT primers and AMV reverse transcriptase (AMV RT kit; Promega, Madison, WI). Genomic DNA and cDNA from the Herford Dominette, the animal used for the current bovine genome sequencing and annotation project [25,26], were also obtained with total RNA isolated using a LeukoLOCK kit (Ambion, Austin TX) at USDA-ARS Fort Keogh, while genomic DNA from Red Angus, Angus, Charolais, Limousin, Brahman crossed with Angus, Gelbvieh, and Angus crossed with Hereford were obtained from semen or leukocytes using standard isolation methods at USDA-ARS Clay Center.

### Real time quantitative-PCR

**Figure 1:**
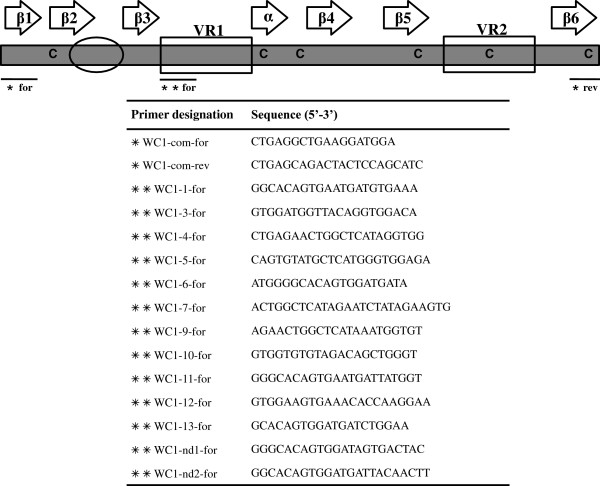
**Schematic representation and primer placement of the predicted structure of WC1 Domain 1.** Secondary structure motifs are indicated above (beta-strand arrows (β1-β6) and an alpha-helix arrow). The RVEVLxxxxW bacterial binding motif is circled. The two regions (VR1 and VR2) that contain most of the sequence diversity between WC1 SRCR domain 1s are boxed. Cysteines are indicated in black [29]. The common forward and reverse primers were designed based on the conserved region and denoted by a single asterisk (*), while the specific forward primers for each WC1 gene are denoted by double asterisks (**).

Genomic DNA from sixteen cattle of ten different breeds was used as template. Real time Q-PCR amplification was done with primers against consensus bovine sequences (i.e. common primers) for all members of each gene family as follows: WC1 SRCR “domain 1” (see Figure 1 for sequence), IFNA (forward 5^′^ ATGGCCCCAGCCTGGTCCTTCC, reverse 5^′^ TCAGTCCTTTCTCCTGAAACTC), IFNB (forward 5^′^ ATGACCTACCGGTGCCTCCTCC, reverse 5^′^ TCAGTCACGGACGTAACCTG), IFNE (forward 5^′^ ATGATTAACAAGGCTTTCTTTG, reverse 5^′^ GCTTTTAAAGCCTGCAGTCG), and IFNW (forward 5^′^ ATGGCCTTCATGCTCTCTCTAC, reverse 5^′^ TCAAGGTGAGTTCAGGTCTCCATC). T cell receptor delta joining gene 1 (TRDJ1) (forward 5^′^ CCTCAACCACAAGAGTCTGTAC, reverse 5^′^ CCAGCTGGGAGTCTGAGATC) and Glyceraldehyde-3-phosphate dehydrogenase (*GAPD*) (forward 5^′^ TTCAACGGCACAGTCAAGG, reverse 5^′^ ACATACTCAGCACCAGCATCAC). The WC1 common primer pair (WC1-com-for and WC1-com-rev) was designed to amplify all WC1 “domain 1” sequences based on the conserved region annotated in a previous study [3] (Figure 1). The GenBank accession numbers of the sequences used for designing the primers in this experiment are as follows: IFNA (NM_001017411.1; *IFNA*), IFNB (NM_174350.1; *IFNB*), IFNE (NM_176891; *IFNE*), and IFNW (NM_174351.1; *IFNW*). The T cell receptor delta joining gene 1 (*TRDJ1*) primers and Glyceraldehyde-3-phosphate dehydrogenase (*GAPD*) primers were described previously [27,28]. Gene numbers among animals were evaluated for differences using ANOVA.

Real-time Q-PCR amplification and analysis were performed using a Stratagene Mx3005P instruments with software version 4.01 (Stratagene, La Jolla, CA). The Q-PCR assays were optimized in terms of Mg^2+^ concentration and the annealing temperature [30]. Q-PCR amplification mixture (25ul) was prepared by using Sybr Premix Ex Taq (TAKARA, Pittsburgh, PA): 20 ng template DNA, 2-fold concentration of premix reagent including Takara Ex Taq™ HS and SYBR® Green I, 0.5ul ROX reference dye, and 1ul of forward and reverse primers (final concentration is 0.5uM for each). Real-time PCR amplification was conducted for 35 cycles, each cycle consisting of denaturation (95°C for 5 sec), annealing (55°C for 20 sec) with a single fluorescence measurement taken at the end of the annealing step, and extension (72°C for 20 sec). After amplification, melting-curve analysis was performed by raising the temperature to 95°C for 1 min, heating the sample at 55°C for 30 sec followed by 95°C for 30 sec. The ΔΔC_T_ method was applied for gene number determination [21]: relative amount of targets = (1 + E)^− ΔΔCT^, where ΔΔC_T_ : ΔC_T_ of the targets − ΔC_T_ of the calibrators, ΔC_T_ of the target: C_T_ of the targets − C_T_ of the reference, and ΔC_T_ of the calibrators: C_T_ of the calibrators − C_T_ of the reference. In this case, the ‘targets’ were bovine WC1 domain 1, bovine *IFNA*, bovine *IFNB*, bovine *IFNW* while the ‘reference’ was bovine *GAPD*, and the ‘calibrators’ were bovine *TRDJ1* and bovine *IFNE*. Real-time PCR products were analyzed on 1% or 1.2% TAE agarose gels, visualized using SYBR Safe (Invitrogen) and cloned into the pCR2.1 vector (Invitrogen) according to the manufacturer’s protocol for sequencing.

### PCR amplification specific for Domain 1

For amplifying each Domain 1 sequence specifically, PCR reactions were performed with PCR Mastermix (Promega, Madison, WI) according to the manufacturer’s instructions and primers were designed based on one of the most variable regions of domain 1 (Figure 1, [3]). The reverse common primer (WC1-com-rev) was designed within a conserved region of the domain 1 of all known WC1 molecules as denoted in Figure 1. The GenBank accession numbers of the expressed gene sequences used for designing the primers in this experiment are as follows: WC1-3 (previously known as archetypal WC1.1; X63723), WC1-nd1 (clone CH525; FJ031216), WC1-nd2 (clone CCnd2; JN998896), while the others (WC1-1, WC1-4, WC1-5, WC1-6, WC1-7, WC1-9, WC1-10, WC1-11, WC1-12 and WC1-13) were annotated in our previous study [3] and shown in Table 1. Cycling parameters for those reactions were 30 sec at 95°C, 1 min at 58°C and 1 min at 72°C for 30 cycles with an expected amplicon size of approximately 200 bp. PCR products were visualized using SYBR Safe (Invitrogen) on 1% or 1.2% TAE agarose gels and isolated after visualization and cloned into the pCR2.1 vector (Invitrogen) according to the manufacturer’s protocol and sent for commercial sequencing (GeneWiz, South Plainfield, NJ).

**Table 1:** Chromosomal location in Btau_3.1assembly and GenBank accession number of WC1 genes

**Gene name**	**cDNA clone**	**GLEAN number**	**GenBank accession number**
WC1-1	CH501 ^a^	GLEAN_13183 ^a^	FJ031186
WC1-2	CCnd1	GLEAN_13182 ^a^	JN998897
WC1-3	CH534 ^a^	GLEAN_13181 ^a^	FJ031191 ^a^
WC1-4	CH496 ^a^	GLEAN_13179 ^a^	FJ031202 ^a^
WC1-5	CH590 ^a^	GLEAN_13176 ^a^	JQ900627 ^a^
WC1-6	CC6	GLEAN_00457/GLEAN_00458 ^a^	JN234380
WC1-7	CC7	GLEAN_00456 ^a^	JN234377
WC1-8	CCnd2	GLEAN_12186 ^a^	JN998896
WC1-9	CH505 ^a^	GLEAN_12191 ^a^	FJ031208 ^a^
WC1-10	CH601 ^a^	GLEAN_12192 ^a^	JQ900628 ^a^
WC1-11	CH486 ^a^	GLEAN_12182/GLEAN_09904 ^a^	FJ031209 ^a^
WC1-12	CC12	GLEAN_09902 ^a^	JN234378
WC1-13	CH504 ^a^	GLEAN_12187 ^a^	FJ031187 ^a^

a. Herzig et al., 2009, BMC Genomics, [3].

### PCR amplification for complete coding sequence

For amplifying the complete coding sequence of WC1 genes, 2 μl of pooled cDNA was used as a template and PCR reactions were conducted using the Elongase Amplification system (Invitrogen) with a final concentration of 1.5 mM Mg^2+^. Based on previous research, forward primers in the signal sequence (WC1atg-for 5^′^ATGGCTCTGGGCAGACACCTCTC) and reverse (WC1groups1,2-rev 5^′^TCAYGAGAAAGTCAYTGKGGATG) primers in the intracytoplasmic tail sequence were designed to amplify all known WC1 transcripts except WC1-11 which required the following primers: forward (WC1atg-for 5^′^ATGGCTCTGGGCAGACACCTCTC) and reverse (WC1group3rev 5^′^-CTACATGGTGCTAAGCTCCACATC) [3]. Cycling parameters were 30 sec at 94°C, 30 sec at 55°C and 5 min 30 sec at 68°C for 35 cycles for all reactions. PCR products were analyzed on 1.2% TAE agarose gels, visualized using SYBR Safe (Invitrogen) and cloned into the pCR-XL vector (Invitrogen) for sequencing.

### Sequence analyses

Sequencing was performed commercially (Genewiz) to verify amplicons. Nucleotide sequences were aligned and consensus sequences were created using Bioedit version 7.0.5.3 [31]. GenBank accession numbers of annotated sequences used for comparisons in analyses are shown in Table 1 as annotated and/or reported in our previous research [3] except archetypal WC1.1 whose GenBank number is X63723. Multiple sequence alignments were performed using clustalw2 ( http://www.ebi.ac.uk/Tools/clustalw2/index.html webcite; [32]) and the default parameters, but manually optimized when necessary, and were visualized using Bioedit [31]. Phylogenetic analyses were performed using deduced amino acid sequences of WC1 domain 1 as indicated. Phylogenetic trees were created using Bayesian analysis in MrBayes3.2 [33]. For Bayesian analysis, 2 runs with 3 cold chains and 1 heated chain each were done. An amino acid mixed model was used to approximate the posterior probabilities of trees. The 90-taxa SRCR domain 1 alignment was run with temperature settings of 0.2 for 830,000 generations. Trees were sampled every 100 generations and the burnin fraction was 0.25. The convergence diagnostic used was the average standard deviation of split frequencies, which were <0.05 (0.01) for the run. Phylograms were visualized using FigTree V1.3.1 (http://tree.bio.ed.ac.uk/software/figtree/).

### Magnetic bead cell sorting

PBMC were stained for surface markers at 4°C for 20 min in PBS with 2 mM EDTA and 0.5% BSA. The anti-WC1 mAb BAG25A (VMRD, Pullman, WA) for WC1.1 epitopes was used for sorting. Cells were then incubated with goat anti-mouse IgM-conjugtated magnetic microbeads (Miltenyi Biotec, Auburn, CA, USA) at 4°C for 20 min. After washing twice, cells were applied to the column following the manufacturer’s instructions. The purity of collected fractions was assessed by flow cytometry and analyzed using FlowJo (Tree Star, Ashland, OR, USA).

## Results

### The WC1 family is composed of thirteen genes

Due to gaps in the bovine genome Btau_3.1 assembly [3,26], we were uncertain whether we had identified the total complement of WC1 genes present. Moreover, the possibility existed that gene number variation occurs among breeds of cattle or individuals within a breed. To address this we adapted Q-PCR to determine WC1 gene numbers in the Hereford Dominette, the reference/donor animal used for the Bovine Genome Sequencing and Annotation project [26], as well as in additional breeds of cattle.

Although considerable repetition of sequence occurs among repeating SRCR domains of WC1 molecules (i.e., b,c,d,e,d’), the most distal SRCR domain (domain 1 which has an “a” pattern [1]) of each known WC1 molecule is unique in terms of structure and sequence relative to all other WC1 domains [3] and coded for by a single exon. Thus, we reasoned that the number of SRCR domain 1 gene exons would be proportional to the WC1 gene number. As controls, bovine *IFNA*, bovine *IFNB*, and bovine *IFNW* genes were evaluated in our system since they are multigene families with known gene numbers [34]. Bovine T cell receptor δ J1 gene (*TRDJ1*) and *IFNE* were both used as calibrators since they are present as single gene copies in the bovine genome [28,34]. Bovine glyceraldehyde-3-phosphate dehydrogenase (*GAPD*) was used as a reference gene for DNA quality [27].

**Figure 2:**
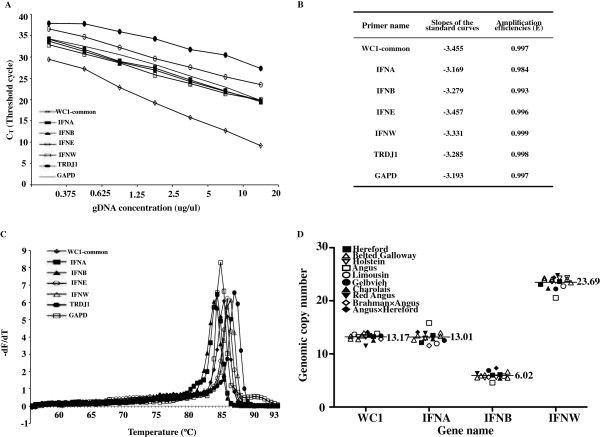
**Q-PCR for WC1 gene number. (A)** The standard curves for primer sets of WC1s (WC1-com) and other control genes. The standard curves were constructed with a series of 10-fold dilution of cDNA derived from ex vivo PBMC. Each standard dilution was amplified by real-time QPCR in duplicate. For each primer set, C_T_ values determined from real-time QPCR were plotted against the logarithm of their known initial gene numbers. A standard curve was generated by linear regression through these points. **(B)** The slopes of the standard curves for primers of WC1s (WC1-com) and other control genes. From the slopes, amplification efficiencies were also determined. **(C)** Primer specificities of WC1s (WC1-com) and other control genes’ primer sets. Confirmation of PCR amplification specificities by melting curve for primer sets of WC1 common (WC1-com), bovine *TRDJ1*, bovine *GAPD*, bovine *IFNA*, bovine *IFNB*, bovine *IFNE*, and bovine *IFNW*. Melting peaks were examined for WC1 common (WC1-com, solid line with empty diamond marker), bovine *TRDJ1* (solid line with solid circle marker), bovine *GAPD* (solid line), bovine *IFNA* (solid line with solid square marker), bovine *IFNB* (solid line with solid triangle marker), bovine *IFNE* (solid line with empty circle marker), and bovine *IFNW* (solid line with empty square marker). **(D)** Gene numbers of bovine WC1 genes and other control genes*.* The breeds of tested cattle are indicated in the figure. The ΔΔC_T_ method was applied for relative quantification. Some breeds contained more than one animal, and each evaluation was performed at least twice, yielding similar results.

Primer sets were designed to amplify all members within a family (*IFNA*, *IFNB* and *IFNW*) and for all known WC1 domain 1 sequences identified to date (see common primers for WC1 in Figure 1). Standard curves for primer sets that amplify all WC1 known genes and for bovine *TRDJ1*, *GAPD*, *IFNA*, *IFNB*, *IFNE*, and *IFNW* were constructed with a range from 20 ug/ul to 0.375 ug/μl (Figure 2A). All curves were linear in the range tested (R^2^ >0.95) in duplicate reactions. The slopes of the standard curves and amplification efficiencies (E) were determined to be in the tested range (Figure 2B) and thus the primers were used for further relative quantification. The specificity for primers was determined by the melting curve analysis to rule out amplification of non-specific PCR products [35]. Figure 2C shows sharp peaks in the fluorescence signal around the melting temperature (T_M_) of the PCR products. In addition, the 2^-ΔΔCT^ relative quantification method requires amplification efficiencies of the target and reference to be approximately equal to be valid [36]. It was observed that the difference between amplification efficiencies of the targets and the reference (bovine *GAPD*) were less than 0.1 which indicates that the amplification efficiencies of the target and reference were similar enough to perform the 2^-ΔΔCT^ relative quantification method.

The results from the relative quantification and the calculated gene numbers are shown in Figure 2D, which were based on amplification efficiencies calculated as described above and the equation (relative amount of target = (1 + E)^− ΔΔCT^ ) described previously [21]. The results for sixteen animals of ten different breeds of cattle (Herford, Belted Galloway, Holstein, Red Angus, Angus, Charolais, Gelbvieh, Limousin, Brahman cross Angus, and Angus cross Hereford) showed a mean gene number of 13.01, 6.02, and 23.69 for bovine *IFNA* (13 expected), bovine *IFNB* (6 expected), and bovine *IFNW* (24 expected), respectively, which are consistent with results in previous studies [26,34]. For bovine WC1 genes we obtained a mean gene number of 13.17 (Figure 2D). According to the obtained Q-PCR results, the number of WC1 genes for some tested cattle (one Holstein and one Red Angus) was less than thirteen. It is possible that those cattle have fewer than 13 WC1 genes, but statistical analysis indicated that the mean number of WC1 genes was thirteen without variation among all the tested individuals and breeds. Thus we conclude that the bovine genome contains thirteen WC1 genes and that this number is consistent among ten breeds of cattle.

### Complete SRCR domain 1 sequences of the thirteen WC1 genes in the donor/reference animal Dominette

We previously annotated thirteen WC1 genes distributed between two regions on bovine chromosome 5 in the bovine genome Btau_3.1 assembly [3]. However, only partial sequences for four of the genes (*WC1-2, WC1-3, WC1-6, WC1-8*) were annotated due to gaps in the genome sequences while some lacked complete transcript sequences [3] (see Table 2 for a summary). Coincident with the annotation, cDNA analysis of material derived from a different animal provided evidence for two additional WC1 domain 1 sequences (designated *WC1-nd1* and *WC1-nd2),* that were not placed in the genome [3] (Table 2). We reasoned that these represented sequences that were not identified as a result of gaps in the genome assembly of Dominette or breed-dependent polymorphisms in WC1 gene sequences since above we report there are only thirteen WC1 genes in all animals evaluated. To distinguish between these two possibilities, Dominette’s genomic DNA as well as cDNA from her PBMC were amplified by primers specific for each of the thirteen WC1 domain 1 sequences available to us including sequences for *WC1-nd2* and *WC1-nd1* which were not present in the genome assembly. The WC1 gene-specific forward primers were based on one of the most variable regions of domain 1 sequences (denoted by double asterisk in Figure 1) while the reverse primer was designed within a conserved region.

**Table 2:** **Available sequence information from the 13 WC1s in previous study**^**a**^

**Gene Name**	**Annotated genomic sequences from Dominette**^**b**^	**RNA transcripts representing the expressed gene sequences obtained from other animals**
WC1-1	+	+
WC1-2	Db7-Dd’11^c^	-
WC1-3	D1^c^	+
WC1-4	+	+
WC1-5	+	+
WC1-6	D1-Dd’11^c^	-
WC1-7	+	-
WC1-8	Dc8-Dd’11^c^	-
WC1-9	+	+
WC1-10	+	+
WC1-11	+^d^	+
WC1-12	+	-
WC1-13	+	+
WC1-nd1	-	+^d, e^
WC1-nd2	-	D1 only^e^

a. Herzig et al., 2009, BMC Genomics, [3].

b. Dominette was the reference animal whose DNA was used by The Bovine Genome Sequencing and Analysis Consortium, [26]; D = domain, and they are described by their pattern type (a-d’) as well as presumed position within the molecule (Domain 1 to 11). + = all 11 domains present in assembled genome or corresponding cDNA obtained.

c. Only partial sequences were obtained due to gaps in the genome sequences.

d. A transcript corresponding to SRCR Domain 1 through the intracytoplasmic region were obtained but it coded for only six extracellular domains, thus it was smaller than the majority of other WC1 molecules [3,14].

e. While transcripts for the SRCR Domain 1 of the proposed genes were found, they were named *WC1-nd1* and *WC1-nd2* because their placement was “not determined” in the genome of Dominette.

**Figure 3:**
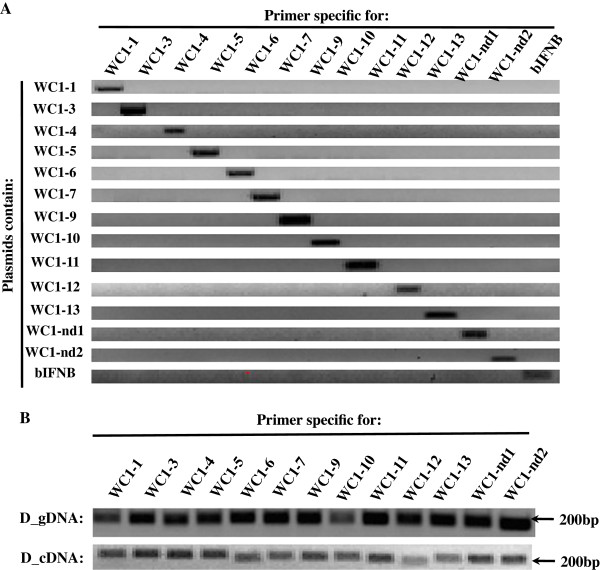
**Specific amplification of each WC1 Domain 1. (A)** Primer specificities of thirteen WC1 specific primer sets. Confirmation of PCR amplification specificities by gel electrophoresis for thirteen WC1 specific primer sets (*WC1-1, WC1-3, WC1-4, WC1-5, WC1-6, WC1-7, WC1-9, WC1-10, WC1-11, WC1-12, WC1-13, WC1-nd1,* and *WC1-nd2*) and the bovine *IFNB* primer pair. Plasmids containing thirteen WC1 domain 1 gene sequences were used as templates in PCR reactions. For each primer set, the identities of the amplified products were confirmed by DNA sequencing analysis. **(B)** Genomic DNA and cDNA evidence for Dominette. PCR analysis was conducted by using genomic DNA and cDNA derived from the reference animal Dominette of the Hereford breed (designed with prefixes of “D_gDNA and D_cDNA”, respectively). Primer pairs for amplification of thirteen WC1 SRCR domain 1 sequences with the specific primer sets tested in (A). For each primer set, the identities of the amplified products were confirmed by DNA sequencing analysis.

To verify the amplification specificity of those primer sets, PCR reactions were performed on plasmids containing each of thirteen WC1 domain 1 sequences. The primer pair for bovine *IFNB* was used as the negative control since bovine *IFNB* is not related to bovine WC1. Each set of thirteen WC1 PCR primers generated a single prominent band with expected size (200bp) when the templates contained its corresponding WC1 gene (Figure 3A), indicating that the thirteen specific WC1 forward primers in conjunction with the common reverse primer amplified only their corresponding domain 1 sequence. This was validated by sequencing the amplicons. In addition, we verified the absence of nonspecific amplification of carrier sequences by PCR-amplification of negative controls (vector without inserts and with unrelated inserts), and the absence of contamination by the PCR-amplification of negative ‘no template’ control (data not shown).

Analysis of PCR products obtained showed that all thirteen known WC1 domain 1 sequences, including that for *WC1-nd1* and *WC1-nd2,* were present in both Dominette’s genomic DNA and cDNA (Figure 3B). Thus, we conclude that W*C1-nd1* and *WC1-nd2* correspond to gaps in the assembled genome and reasoned that they might represent the missing *WC1-2* and *WC1-8* domain 1 sequences.

### Generating templates to obtain complete coding sequences for all thirteen WC1 genes

**Figure 4:**
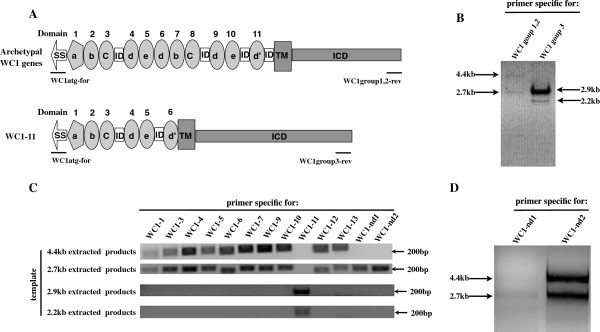
**Amplification of full-length transcripts for WC1s. (A)** Primers designed for complete coding sequences of all thirteen WC1 genes. Schematic representations of the molecular forms of archetypal WC1 genes and *WC1-11* with primer placement indicated. The WC1 common forward primer (WC1atg-for) for complete coding sequences was designed based on the conserved region in the signal sequences, while the reverse primers (WC1group1,2-rev and WC1group3-rev) were based on the end of the 3^′^ coding sequences. Abbreviations are as follows: ID, inter-domain sequence; TM, transmembrane region; ICD, intracytoplasmic domain. **(B)** cDNA evidence for WC1 genes. Primer pairs WC1atg-for/WC1group1,2-rev (designed for WC1group1,2) and primer set WC1atg-for/WC1group3-rev (designed for WC1group3) were used to amplify all the complete coding sequences of WC1 transcripts as described in the previous study [3]. **(C)** Confirmation of complete coding sequences for *WC1-nd1* and *WC1-nd2*. Four different templates used in PCR for all thirteen WC1 domain 1 specific primer pairs are indicated in the left part of each gel. **(D)** Agarose gel electrophoresis evidence for complete coding sequences of *WC1-nd1* and *WC1-nd2.* Complete coding sequences of *WC1-nd1* and *WC1-nd2* amplified by primer pairs of specific forward primers and common reverse primers (WC1group1,2-rev). The cDNA isolated from sorted WC1.1^+^ γδ T cells was used as a template. Gel electrophoresis of the PCR products was performed on 1% agarose gel. For each primer set, the identity of the amplified products was confirmed by DNA sequencing analysis.

**Figure 5:**
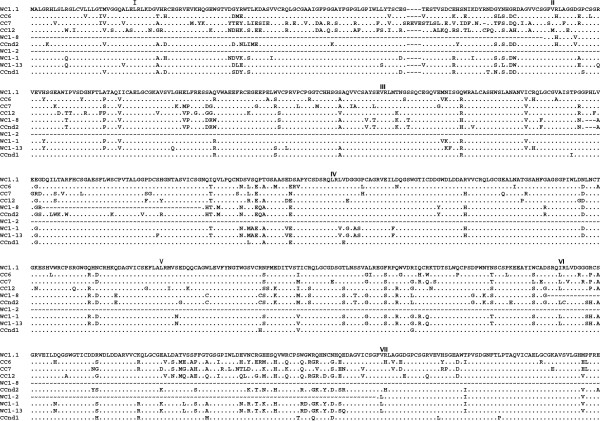
**Complete sequences of cDNA clones CC6 *(WC1-6), *CC7 *(WC1-7), *CC12 *(WC1-12), *CCnd1 *(WC1-nd1) *and CCnd2 *(WC1-nd2).*** Deduced amino acids sequence from the coding sequences of *WC1-1*, *WC1-2*, *WC1-8*, and *WC1-13*, and the archetypal WC1 (Wc1.1) sequence were aligned using ClustalW2 and the default parameters and refined manually. GenBank accession numbers for amino acid sequences used for comparison are described in Materials and Methods. Identities are indicated by dots (.), gaps resulting from the alignment are indicated by tildes (~), gaps resulting from lack of genomic sequence (when the gaps were found adjacent and not within a coding region) are indicated by dashes (-). SRCR domains are indicated in Roman numerals and the transmembrane region is shown underlined for archetypal WC1 sequence. Continued in Figure 6.

**Figure 6:**
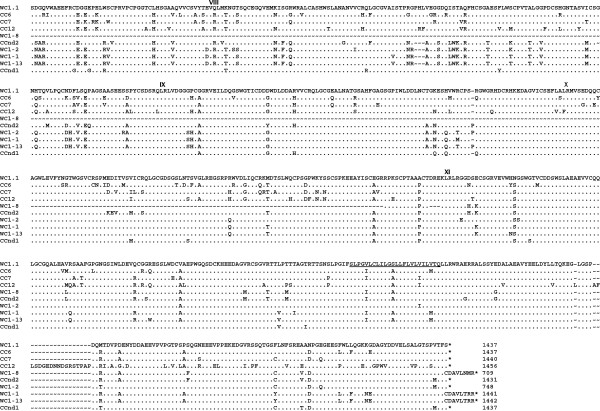
**Complete sequences of cDNA clones CC6 *(WC1-6), *CC7 *(WC1-7), *CC12 *(WC1-12), *CCnd1 *(WC1-nd1) *and CCnd2 *(WC1-nd2)*, continued*.*** Deduced amino acids sequence from the coding sequences of *WC1-1*, *WC1-2*, *WC1-8*, and *WC1-13*, and the archetypal WC1 sequence (WC1.1) were aligned using ClustalW2 and the default parameters and refined manually. GenBank accession numbers for amino acid sequences used for comparison are described in Materials and Methods. Identities are indicated by dots (.), gaps resulting from the alignment are indicated by tildes (~), gaps resulting from lack of genomic sequence (when the gaps were found adjacent and not within a coding region) are indicated by dashes (-). SRCR domains are indicated in Roman numerals and the transmembrane region is shown underlined for archetypal WC1 sequence.

To attempt to obtain the complete coding sequences, intracytoplasmic tail sequences were aligned and a common intracytoplasmic tail sequence primer was designed along with a forward primer in the 5^′^ signal sequence (Figure 4A). These primers amplified material of approximately 4.4kb (as described previously [3]) using cDNA from Dominette’s ex vivo PBMC (Figure 4A). In addition there was a smaller major band of 2.7kb (Figure 4B). Because the intracytoplasmic tail sequence of the gene we previously designated *WC1-11* was so different from the others [3], a separate reverse primers had to be designed (Figure 4A); when used it amplified two bands of 2.9kb and 2.2kb (Figure 4B). All four bands were extracted from the gels and evaluated as templates from which to amplify each of the thirteen WC1 domain 1’s using gene-specific primer sets. PCR products for ten of the WC1 genes were obtained using the 4.4 kb material with *WC1-nd1*, *WC1-nd2* and *WC1-11* being the exceptions (see Figure 4C for those amplified). The 2.7kb bands proved to contain templates for twelve WC1 genes with *WC1-11* being the one exception as expected (Figure 4C). The *WC1-11* gene sequences were associated with both the 2.9kb and 2.2kb bands, also as expected. Because we were not able to amplify *WC1-nd1* and *WC1-nd2* from the 4.4 kb band, we enriched for WC1 transcripts by sorting for WC1.1^+^ γδ T cells. The cDNA derived from this was amplified with common primers as described above and the 4.4 kb PCR products was found to contain transcripts corresponding to *WC1-nd1* and *WC1-nd2* domain 1s (Figure 4D, Figure 5, and Figure 6).

The results also confirmed and extended our previous observations that most WC1 transcripts display alternative splicing of coding exons given that domain 1 sequence of all 13 WC1 genes could be amplified from the smaller (as well as the larger) bands [3].

### Complete coding sequences for the annotated *WC1-6, WC1-7 and WC1-12* genes

To confirm complete sequences for transcripts (i.e. cDNA clones) representing the complete coding sequences of *WC1-6, WC1-7 and WC1-12*, the template material described in the previous section was used. Forward primers specific for domain 1’s of *WC1-6, WC1-7 and WC1-12* (Figure 1) were combined with the common reverse primer in the intracytoplasmic tail (Figure 4) [3] and used to successfully amplify the material extracted from the 4.4 kb band described above. The PCR products were sequenced and the deduced amino acid sequences were aligned with the archetypal WC1.1 sequence (now known as *WC1-3*) (Figure 5 and Figure 6). The percent identities were calculated based on those alignments (Table 3) and showed that indeed these represent the complete coding sequence for *WC1-*6, *WC1-7* and *WC1-12*.

**Table 3:** **cDNA evidence for transcription of *WC1-6*, *WC1-7*, *WC1-12*, *WC1-nd1 *and *WC1-nd2***^**a**^

	**cDNA clone**^**b**^				
**Gene Name**	**CC6**	**CC7**	**CC12**	**CCnd1**	**CCnd2**
WC1-1	94	62	71	96	90
WC1-2^c^	88	63	65	**97**	91
WC1-3	93	63	69	93	91
WC1-4	64	96	61	65	62
WC1-5	93	66	70	95	93
WC1-6	**99**	66	68	92	90
WC1-7	66	**98**	62	65	62
WC1-8^c^	90	62	66	92	**97**
WC1-9	64	96	62	63	61
WC1-10	65	58	74	66	66
WC1-11	70	62	72	67	67
WC1-12	68	62	**98**	70	66
WC1-13	97	65	70	94	91

a. Percent identity is shown for the deduced amino acid sequences of WC1 genes and cDNA clones.

b. Bold values indicate the highest level of identity and thus classification of the cDNA clone as a particular WC1 gene.

c. The annotation of these genes (*WC1-2* and *WC1-8*) was found to be partial due to gaps in the genome assembly but shown here to be represented by *WC1-nd1* and *WC1-nd2*, respectively.

#### WC1-nd2 is WC1-8

Using the templates generated above and the forward primer specific for *WC1-nd2* domain 1 sequence (Figure 1)*,* coding sequence for *WC1-nd2* was obtained from the 4.4 kb amplified material. This sequence was compared to the partially annotated *WC1-8* sequence and found to be identical. Thus, despite the fact that domain 1 and 2 sequence for *WC1-8* was unavailable due to gaps in the genome (Table 2, [3]) we have re-classified *WC1-nd2* as *WC1-8* and henceforth will refer to it as such*.*

#### WC1-nd1 is WC1-2

**Figure 7:**
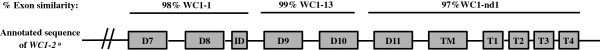
**Structure of the mis-assembled WC1-2.** Schematic representation of *WC1-2* with the eleven exons annotation as in a previous study (not shown to scale, [3]). The gap adjacent to the coding region of *WC1-2* in the Btau 3.1 assembly is indicated by double slashes (//). Abbreviations are as follows: D, SRCR domain; ID, inter-domain sequence; TM, transmembrane region; T, intracytoplasmic tail exon numbers.

Using the templates generated above and the forward primer specific for *WC1-nd1* domain 1 sequence (Figure 1)*,* coding sequence for *WC1-nd1* was obtained from the 4.4 kb amplified material and compared to partially annotated WC1 genes. We found that it corresponded to *WC1-2’s* domain 11 (d’ pattern), transmembrane and intracytoplasmic tail sequences with 97% similarity (Figure 7). However the annotated *WC1-2* domain 7 and 8 had 99% similarity with *WC1-1* while domains 9 and 10 were 99% identical to *WC1-13*. This suggested that the gene previously referred to as *WC1-2* was a concatemer of mis-assembled genes (Figure 7). To probe for the existence of *WC1-2* sequence as annotated, we attempted to amplify *WC1-2* transcripts by designing three sets of primer pairs that spanned D7 to D9, D7 to the intracytoplasmic tail, and D9 to the intracytoplasmic tail based on sequence of the annotated *WC1-2* sequence [3]. The primers were shown to be functional by pairing with other known primers but they could not amplify across the regions that corresponded to different WC1 gene sequences (data not shown). Since WC1^+^ γδ T cell subsets respond to different activation stimuli [9,37], PCR reactions were performed with pooled cDNA from ex vivo, ConA-activated and *Leptospira*-activated PBMC in order to maximize the number of WC1 transcripts available. Since cDNA evidence corresponding to *WC1-2* as assembled and annotated was not found it confirmed our hypothesis that the annotated *WC1-2* was derived from an assembly anomaly. We suggest that *WC1-nd1* should be instead classified as *WC1-2* based on the evidence that one extracellular domain as well as the transmembrane and intracytoplasmic tail sequences corresponded to the annotated *WC1-2* sequence and our evidence that only thirteen WC1 genes exist.

### WC1 genes in other breeds of cattle

**Figure 8:**
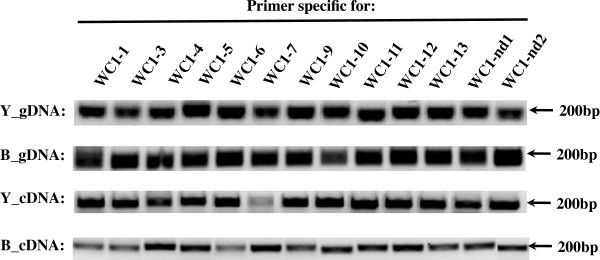
**Genomic DNA and cDNA evidence for the 13 WC1 genes in two bovine breeds.** PCR analysis was conducted by using genomic DNA and cDNA derived from cattle of two different breeds (Belted Galloway, designed with prefixes of “Y_gDNA and Y_cDNA”, respectively; Holstein, designed with prefixes of “B_gDNA and B_cDNA”, respectively). Primer pairs for distinguishing thirteen WC1 domain 1s were thirteen WC1 specific primer sets. For each primer set, the identities of the amplified products were confirmed by DNA sequencing analysis.

**Figure 9:**
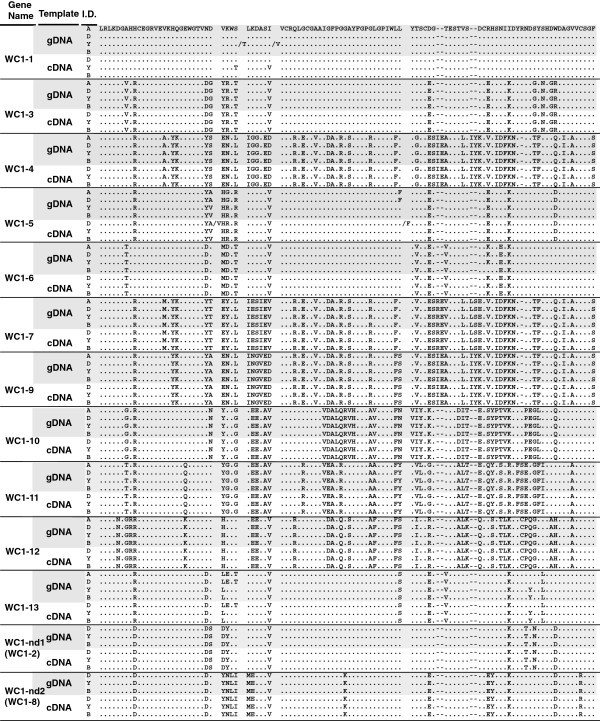
**Comparison of genomic and cDNA WC1 domain 1 sequences for different bovine breeds.** WC1 domain 1 deduced amino acid sequences were aligned with ClustalW2 using the default parameters and visualized with Bioedit. Analysis includes all non-redundant genomic sequences (denoted by gray shades) and cDNA sequences. Annotated sequences were designed with a prefix of “A”. Sequences obtained from individuals of three breeds (Hereford, Belted Galloway, and Holstein) were designed with prefixes of “D”, “Y”, and “B”, respectively. Identities are indicated by dots (.). Gaps resulting from the alignment are indicated by tildes. Amino acids representing heterozygous alleles are separated by a *slash*.

To determine whether the same thirteen known WC1 domain 1 gene sequences are conserved in other breeds, genomic DNA and cDNA from two other breeds of cattle (Belted Galloway and Holstein) were amplified using the thirteen WC1 gene-specific primers. Each animal showed products corresponding to all thirteen WC1 domain 1 sequences (Figure 8). To determine sequence similarity among individuals and breeds for the thirteen domain 1 sequences we used domain 1 common forward and reverse primers (Figure 1) to amplify genomic DNA and cDNA from three different breeds (Hereford, Belted Galloway, and Holstein). A total of 160 clones were sequenced until a complete set of thirteen different domain 1 sequences for each breed was obtained. The deduced amino acid sequences were aligned and showed complete identity in the majority of cases and a maximum of three amino acid differences in the least similar (Figure 9). Those amino acid differences among animals of different breeds (such as the *WC1-13* domain 1 genomic sequences from Hereford and Belted Galloway) suggest that a modest number of individual or breed-related polymorphisms are present (Figure 9). Allelic polymorphisms were also found since an individual animal had two different transcript sequences in some instances (such as the *WC1-1* derived from a Belted Galloway) (Figure 9).

**Figure 10:**
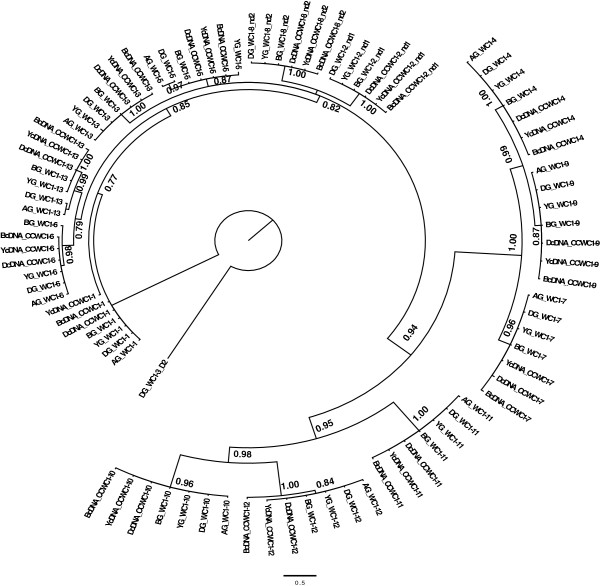
**Phylogenetic tree of WC1 domain 1 sequences.** The evolutionary history of 90 taxa was inferred using Bayesian analysis in MrBayes3.2 [33]. Annotated sequences were designed with a prefix of “A”. Sequences obtained from individual animal of three breeds (Hereford, Belted Galloway, and Holstein) were designed with prefixes of “D”, “Y”, and “B”, respectively. Genomic DNA and transcripts sequences were designed with prefixes of “G and cDNA”, respectively. Markov chain Monte Carlo analysis was performed for 830,000 cycles, using 2 runs of 4 chains each, a temperature setting of 0.2, and an amino acid mixed model to approximate the posterior probabilities of trees, shown at branch nodes. The average standard deviation of split frequencies was 0.01, which was diagnostic of convergence at <0.05. Bootstrap values >70 are shown.

A phylogram generated based on deduced amino acid domain 1 sequences (Figure 10) further confirmed that each of the domain 1 sequences clustered with its corresponding annotated sequence from the reference animal. Of 160 sequences from three breeds of cattle, no additional sequences beyond the thirteen described WC1 genes were found. Thus we conclude that polymorphisms of WC1 genes are rare in *Bos taurus*. Moreover, those gene sequences derived previously from *Bos indicus* cattle (i.e. *WC1-3*, previously WC1.1, and *WC1-4*, previously WC1.2) were also conserved [7].

## Discussion

**Figure 11:**
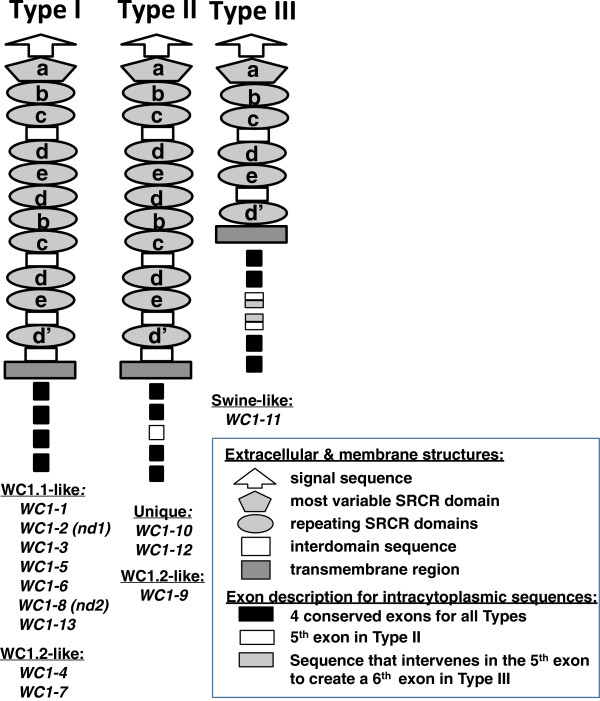
**Schematic representations of three types of WC1 molecules.** The WC1 genes corresponding to the schematics are indicated. WC1^+^ γδ T cells were defined based on mAb reactivity as WC1.1^+^, WC1.2^+^, and WC1.3^+^ wherein the WC1.3^+^ population is a subpopulation of WC1.1^+^ cells [7]. According to their domain 1 sequence similarities with bovine archetypal WC1.1 (*WC1-3*), archetypal WC1.2 (*WC1-4*) or swine WC1 domain 1 sequences, WC1 genes were further classified into groups: WC1.1-like, WC1.2-like, unique, and swine-like. Among WC1.1-like WC1 genes, *WC1-8* (*nd2*) represents the gene product which reacts with the anti-WC1.3 mAb (Chuang and Baldwin, unpublished data) and which is different from the WC1.3 gene sequence reported by Wijngaard et al. [7]. No WC1 genes have the SRCR domain 1 sequences reported by Wijngaard et al. [7]; instead, the most similar sequence is that of SRCR domain 6 of *WC1-4* and *WC1-9*, and we thus suggest part of the published WC1.3 sequence is erroneous.

The complexity of the WC1 multi-gene family in cattle has been resolved in this study: thirteen functional genes were found associated with ten different breeds of animals. The question of gene number variation among cattle was addressed by adapting Q-PCR for quantification. While the result was consistent with our previous WC1 gene annotation undertaken as part of the Bovine Genome Sequencing and Annotation Consortium [3], errors existed in the annotation due to incomplete or mis-assembly of the genome and those were corrected herein. The confirmation of thirteen WC1 genes corresponds reasonably well to the estimate derived by Southern blot, which suggested nineteen genes [14], and another study from our group suggesting thirteen genes based on the number of unique intracytoplasmic tail transcripts obtained [15]. However, it is fewer than the fifty WC1 genes predicted for sheep by Southern blotting [12,13]. Recently, we obtained evidence that sheep have twice the number of WC1 genes as cattle (Kim, Chen and Baldwin, unpublished data). Sequences of SRCR domain 1, the most divergent among the WC1 domains, showed that the domain 1 sequence for an individual gene is highly conserved among breeds, with zero to three amino acids differences found per gene. Despite these differences, phylograms confirmed that the evolutionary divergence between individual WC1 genes was still greater than the divergence among animals for a particular gene. This suggests that the array of WC1 genes has been conserved for diverse functions. Also, we now conclude that there are three distinct WC1 molecular forms based on variation in the number of extracellular domains and intracytoplasmic tail sequences including their signaling motifs (Figure 11). These differences in the molecular structure of members of this multi-gene family have implications regarding ligand binding capacity and its signaling outcomes, which would be consistent among animals.

The conservation of WC1 gene sequences among animals and the number of family members is similar to those characteristics of other pattern recognition receptor (PRR) families. It has been proposed that under natural selection pressure, closely related non-rearranging immunoreceptors found on lymphocytes and antigen-presenting cells diversify in response to multiple ligands, such as bacterial and viral pathogen-associated molecular patterns (PAMPs) [38,39]. PRR’s that recognize PAMPs include Toll-like receptors (TLRs) and the functionally similar but structurally distinct NOD-like receptors (NLRs). Individual TLRs and NLRs specifically recognize individual PAMPs, but also act together to recognize diverse microorganisms, initiating a range of host defense mechanisms [40,41]. The TLR family consists of 10 functional genes in humans [42], 12 in mice and ten in cattle [38] while NLRs [39] have 22 genes in humans and 34 in mice [43]. Two other multi-gene families expressed on NK and γδ T cells are the C-type lectin-like Ly49 family [44-46], which is encoded by 15 functional genes in mice [47,48] but only a single related gene in humans and cattle [49,50], and the killer-IG-like receptor (KIR) family [50-52] which underwent rapid repeated gene duplication in humans and cattle and has 4-14 genes depending upon the individual [47,50,51,53,54]. The ligands for Ly49 and KIR are comprised of a large family [50,55], including MHC class I-related molecules, that are rapidly evolving to evade the immune system. For example, infection of mice with murine cytomegalovirus (MCMV) caused the outgrowth of MCMV mutants which allowed the virus to escape recognition by the activating NK-cell receptor Ly49H [56].

Thus, we hypothesize that the WC1 family also expanded to keep pace with immune challenges from multiple pathogenic microorganisms and may be particularly important to γδ T cells given that the TCR γ gene usage of WC1^+^ cells is restricted [11]. Evidence to support this comes from our and other’s studies showing that the expression of particular WC1 molecules defines subpopulations of bovine WC1^+^ γδ T cells that differ in their response to pathogens [9,37] and irradiated/stressed autologous monocytes [9]. In addition, shRNA-mediated selective reduction of WC1 expression by γδ T cells decreases γδ T cell response to *Leptospira*, supporting the hypothesis that WC1 proteins function as PRRs [29]. Moreover, some members of the SRCR superfamily have been shown to bind PAMPs via interactions with one or multiple SRCR domains. That is, the group B SRCR molecules CRP-ductin, Spα and CD6 specifically bind to the bacterial products lipoteichoic acid (LTA) and lipopolysaccharide (LPS) [57-59] and DMBT1 binds to selected bacteria through a RVEVLxxxxW motif in most of its SRCR domains [60]. Recently, we have localized *Leptospira*-binding activity to five of the eleven individual SRCR domains of specific WC1 molecules (Hsu and Telfer, unpublished data).

With regard to correcting errors in the previous assembly and annotation, here we found that *WC1-nd1* and *WC1-nd2,* the two WC1 transcripts that did not correspond to sequences in the Btau_3.1 genome assembly in our previous study [3], are indeed present in the genome and are transcribed by Dominette. The inability to identify corresponding genomic sequence for *WC1-nd2* resulted from gaps in the assembly. That is, *WC1-8* was a partial sequence with no WC1 domain 1 sequence available, but we show here that it corresponds to *WC1-nd2* by analyzing the complete transcript sequence. The second major error was regarding a gene previously annotated as *WC1-2,* which was found to be a concatamer of mis-assembled SRCR domains corresponding to domains of *WC1-1, WC1-13,* and *WC1-nd1.* Using genomic DNA and cDNA from Dominette, we found that the most membrane-proximal SRCR domain, the transmembrane region and the intracytoplasmic tail sequences of the previously annotated *WC1-2* corresponded to our unplaced to *WC1-nd1* sequence. Thus, *WC1-nd1* has been assigned as *WC1-2,* completing the panel of thirteen complete coding sequences for WC1 genes. In an attempt to further confirm our conclusions, we searched for WC1 sequences in the more recently released assemblies Btau_4.0 and UMD3 but found them to be less informative. WC1 coreceptors are unique to T cells of “γδ T cells high” species [2] including cattle [14] but not found for “γδ T cells low” species, such as human or mice [2]. Thus, the gaps regarding the WC1 coding region in assemblies Btau_3.1, Btau_4.0 and UMD3 may be a consequence of the absence in the human genome which was used for scaffolding the bovine genome.

WC1 gene products classified as Type I and II have eleven extracellular SRCR domains organized in the domain pattern of a-[b-c-d-e-d]-[b-c-d-e-d’] [3] (Figure 11) in comparison to the one Type III gene (*WC1-11*) which codes for six extracellular SRCR domains (a-[b-c-d-e]-[d’]) and is closely related to the swine WC1 gene [3]. The complete sequences of the two new WC1 genes indicates that they can be classified as Type I WC1 genes based on their eleven extracellular domains and intracytoplasmic tail sequences coded for by four exons [3]. Thus, the Type I group is the largest with nine WC1 members. WC1 molecules that contain eleven extracellular domains with two repeating cassettes of highly related (domains b-c-d-e-d) may be advantageous for ligand binding. It is possible that the shorter molecule, WC1-11, represents an ancestral form although it is interesting that the alternatively spliced variants of the eleven-domain WC1 molecules [3] makes them similar in size to WC1-11 and that these shorter splice variants are found associated with stimulated cells (Chen et al., unpublished data). This perhaps represents a regulatory mechanism for dampening the response to pathogens. Alternatively, the shorter forms being more similar in size as the TCR may function to co-bind pathogens more efficiently since γδ TCRs do not see processed antigens but are able to interact with pathogens in a manner more akin to that of antibodies [61]. It will be important to develop an understanding of the functionality of those alternative splice forms and their affects on the immune response of γδ T cells in future studies. Finally, it is notable that in all alternative spliced transcripts the extracellular SRCR domain apposed to the membrane is always d’ as it is in full-length molecules, suggesting that the d’ may be structural. It might facilitate co-clustering of WC1 molecules with the TCR since we have shown that both associate with lipid rafts following cell activation [15].

Differences in the intracytoplasmic tails likely play an important role in signal transduction. Type II WC1 molecules have a “long tail” molecular form, with fifteen or more amino acids encoded by an additional (5^th^) exon [3] (Figure 11). Type III contains a very long intracytoplasmic domain resulting from a 6^th^ exon coding for amino acids inserted into the sequence coded for by the middle exon (the 5^th^ exon) of Type II WC1 genes [3] (Figure 11). Short and long tails are also found with other immunoreceptor families: KIR and NKG2D. Activating KIRs have short cytoplasmic tails with ITAMs that pair with DAP12/KARAP; inhibitory KIRs possess long cytoplasmic tails with ITIM motifs [62]. NKG2D long form tails associate with DAP10 [63], while the short form [64] associates with DAP10 or DAP12. The adaptor determines the outcome of signaling following ligand binding [63,64]. A signaling role for the most common WC1 tail sequence, which is the shortest, is shown by the requirement for phosphorylation of the second tyrosine for transmission of signaling through the TCR [4]. It is notable that three gene products, *WC1-4, WC1-7*, and *WC1-9*, all have highly similar extracellular domains, possibly recognizing the same ligands, but the intracytoplasmic tails of *WC1-4* and *WC1-7* are archetypal (or short) while the tail of *WC1-9* is longer as illustrated in Figure 11. This may indicate that cells bearing *WC1-4* or *WC1-7* vs. *WC1-9* have different functional outcomes even if they bind the same ligands consistent with the paired receptor hypothesis for KIR molecules [62]. The signaling role for the other intracytoplasmic sequences of WC1 molecules is under investigation.

## Conclusion

Using Q-PCR to quantitate gene number, we showed that the WC1 immunoreceptor family comprises thirteen genes in the bovine genome, without variation in number among ten cattle breeds tested. Moreover, conservation of sequences for the thirteen WC1 genes existed among breeds. We found that all thirteen WC1 molecules fit into the three distinct molecular forms we previously described. While it has already been shown that functionally distinct subpopulations of bovine WC1^+^ γδ T cells can be defined by the expression of particular WC1 molecules, future studies need to address the significant questions of the signaling potential of each type of WC1 molecule in γδ T cell responses and the identification of ligand-binding domains in the various WC1 molecules. WC1 co-receptors on γδ T cells may be a type of PRRs on nonconventional T cells that participate with the TCR for maximal cell activation. Understanding the mechanism of activation of nonconventional γδ T cells that serve to bridge between innate and adaptive immune response might be exploited for efficacious vaccine design to improve human and domesticated animal health.

## Authors’ contributions

CC carried out the molecular studies, the sequence analyses and drafted the manuscript. CH helped to provide annotation data and participated in the design of the study. AL provided the genomic DNA and cDNA of the reference animal Dominette and revised the manuscript. KJ and MT provided genomic DNA from seven additional breeds of cattle and participated in data interpretation and manuscript editing. JT and CB participated in the design of the study, interpretation of data, securing funding for the study and helped draft the manuscript. All authors read and approved the final manuscript.
